# The Evolution of Classical Spiro-OMeTAD: Synthesis of Arylamine Endcapped Indenone Spirofluorene

**DOI:** 10.3389/fchem.2022.898320

**Published:** 2022-05-31

**Authors:** Shihui Liu, Xiaoqing Yi, Hao Wang, Tao Ye, Kui Wang, Wei Cao, Jing Guan, Ruiqing Fan, Yulin Yang, Sue Hao, Debin Xia

**Affiliations:** ^1^ Department of Organic Chemistry, College of Pharmacy, Harbin Medical University, Harbin, China; ^2^ MIIT Key Laboratory of Critical Materials Technology for New Energy Conversion and Storage, School of Chemistry and Chemical Engineering, Harbin Institute of Technology, Harbin, China; ^3^ State Key Laboratory of Urban Water Resource and Environment, Harbin Institute of Technology, Harbin, China

**Keywords:** spirofluorene, spiro-OMeTAD, indenone, organic semiconductor, polycyclic aromatic hydrocarbons

## Abstract

Spiro-OMeTAD is the well-known hole transporting material (HTM) in perovskite solar cells. In this work, its derivatives, namely four D-A shaped triphenylamine or biphenylamine endcapped indenone spirofluorene (SFD-TPA, SFD-OMeTPA, SFD-TAD, and SFD-OMeTAD), were designed and synthesized. With the introduction of electron-donating moieties and the extension of conjugation length, a series of changes in photophysical and electrochemical properties could be detected. Notably, in comparison with the optical gap (2.96 eV) of the reported spiro-OMeTAD, SFD-OMeTAD presents an optical gap as low as 1.87 eV. Moreover, density functional theory simulations were employed to further investigate their geometric and electronic structures. Finally, steady-state photoluminescence measurements proved the efficient charge separation and collection processes at the perovskite/HTM interface. It can be predicted that all four compounds with enhanced sunlight absorption capability and suitable frontier energy levels can be used as hole-transporting materials for perovskite solar cells.

## Introduction

Perovskite solar cells (PSCs) have attracted considerable attention as next-generation energy sources because of their numerous advantages, such as facile processing, prominent power conversion efficiency (PCE), and relatively low fabrication cost (Li X. et al., 2016; Lee et al., 2016; Ge et al., 2018; Shang et al., 2018; Abuhelaiqa et al., 2019; Jiang A. et al., 2019; Bai et al., 2019). Hole-transporting materials (HTMs) are always required to construct high-efficiency PSCs (Stranks and Snaith, 2015; Gangala and Misra, 2018; Wang et al., 2018; Yu et al., 2019). The most commonly used HTM in record-breaking PSCs is 2, 2′, 7, 7′-tetrakis-(N,N-di-p-methoxyphenylamino)-9,9′-spirobifluorene (spiro-OMeTAD) (Li et al., 2015; Ma et al., 2015; Seo et al., 2016; Wang et al., 2016; Xu et al., 2017; Jiang Q. et al., 2019). However, doping lithium bis-(trifluoromethanesulfonyl)imide salt (LiTFSIA) and 4-tertbutylpyridine (TBP) into spiro-OMeTAD is essential to enhance carrier transporting mobilities (Zhou et al., 2018). As a consequence, the stability of PSCs is deceased due to the hydrophilic properties of these dopants.

Donor-acceptor (D-A) type small molecules can be regarded as a good candidate for HTMs because the D-A molecular backbone features intramolecular charge transfer (ICT) characteristics and a high dipole moment, which could induce self-doping and a built-in potential to boost charge extraction (Liu et al., 2015; Steck et al., 2015; Bi et al., 2016; Kim et al., 2016; Liu et al., 2016; Xu et al., 2018; Mai et al., 2021; Zhang et al., 2021). Li et al. reported a triphenylamine (donor) and tricyanovinylene (acceptor) substituted dipolar chromophore (BTPA-TCNE) to serve as an efficient dopant-free HTM for PSCs in 2016 (Li Z. et al., 2016), generating a promising PCE of ∼17.0%. This result outperforms the control devices using doped spiro-OMeTAD HTM. Recently, Guo et al. reported two novel D-A type HTMs with phenylamine groups as the donor units and imide-functionalized thiophene as the acceptor units (Wang et al., 2019). The dopant-free PSCs achieved a remarkable efficiency of 21.17% with negligible hysteresis and superior thermal stability and long-term stability under illumination, which breaks the long-time standing bottleneck in the development of dopant-free HTMs for highly efficient inverted PSCs.

**FIGURE 1:**
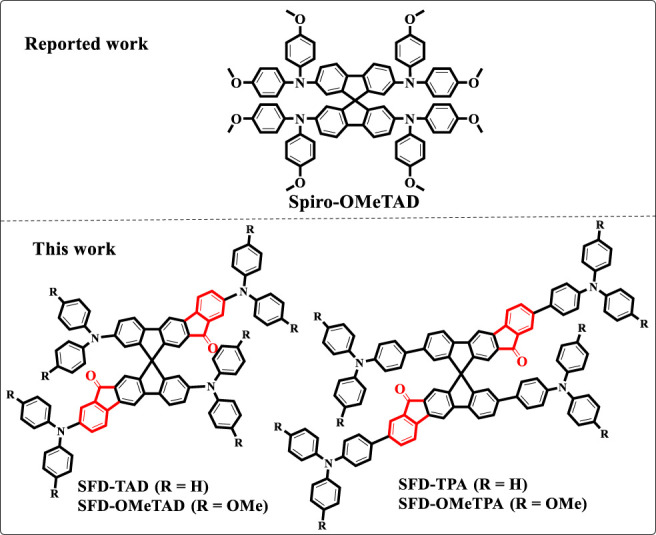
Chemical structures of Spiro-OMeTAD, SFD-TPA, SFD-OMeTPA, SFD-TAD, and SFD-OMeTAD.

Inspired by these works, we report four novel D-A type HTMs as shown in Figure 1, using indenone spirofluorene as acceptor and arylamines as donors based on the following considerations: (1) the extension of conjugation area based on classic spiro core spiro-OMeTAD, which is better for property comparison; (2) nonplanar molecular configuration and large steric hindrance can effectively inhibit π−π aggregation and charge recombination between molecules, thereby promoting hole extraction from the perovskite layer (Pham et al., 2017; Zimmermann et al., 2017; Pham et al., 2018); (3) the rigid structural characteristics of the molecules have a relatively high glass transition temperature, favoring uniform thin film formation (Azmi et al., 2018; Jeon et al., 2018; Liu et al., 2018); (4) carbonyl group as a Lewis base can passivate Pb^2+^ defects on the surface of perovskite, reduce the activity, and improve its humidity stability (Zhang et al., 2018; Chen et al., 2019; Zou et al., 2019); (5) the N atom on the triarylamine has a strong electron donating ability, which is easily oxidized to generate a cationic radical, enabling a high hole mobility (Lin et al., 2003; Zhao et al., 2019). The novel HTMs were characterized by NMR, UV-Vis, and cyclic voltammetry spectroscopy. Steady-state photoluminescence (PL) was also employed to evaluate the hole extraction capability of the perovskite/HTMs interface.

## Results and Discussion

**SCHEME 1:**
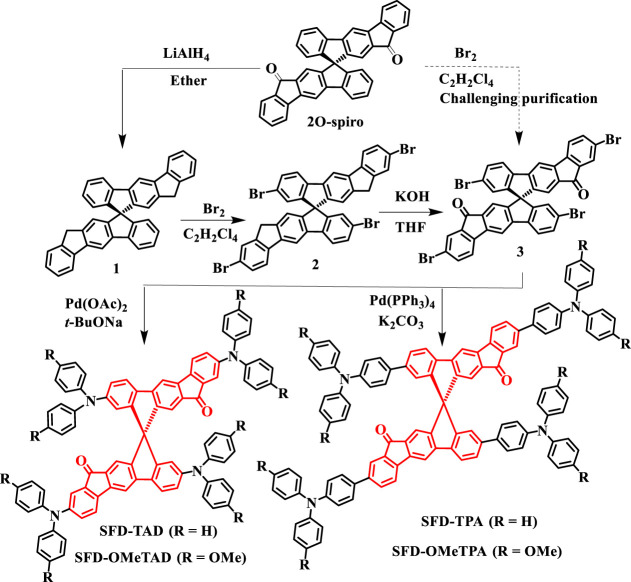
Synthesis route of spiro-OMeTAD derivates.

The detailed synthetic routes for SFD-TPA, SFD-OMeTPA, SFD-TAD, and SFD-OMeTAD are shown in Scheme 1. 12H,12′H-10,10′-spirobi [indeno [2,1-b]fluorene] (2O-spiro) was used as starting material, which was synthesized according to our previous report (Xia et al., 2015). First, employing a bromine reagent, regioselective bromination at the *α* positions of 2O-spiro was attempted in C_2_H_2_Cl_4_. Tetrabrominated intermediate 2,2′,8,8′-tetrabromo-12H,12′H-10,10′-spirobi [indeno [2,1-b]fluorene] (**3**) could be obtained together with five-fold brominated by-products. The mixture was tough to be purified due to its poor solubility and similar polarity. An alternative synthesis strategy was developed. Using LiAlH_4_, 2O-spiro could be reduced to compound **1** in 72% yield. Thereafter, compounds **2** and **3** were prepared *via* bromination and oxidation reaction in yields of 15% and 47%, respectively. Finally, electron-donating group arylamines, as terminal moieties, were covalently bonded to **3** *via* four-fold Suzuki coupling reactions or Buchwald Hartwig cross-coupling reactions. SFD-TPA, SFD-OMeTPA, SFD-TAD, and SFD-OMeTAD were purified using silica gel column chromatography in yields of 40%, 42%, 38%, and 42%, respectively. The structures of the target molecules were fully characterized by ^1^H NMR, ^13^C NMR, and Maldi-TOF-Mass.

**FIGURE 2:**
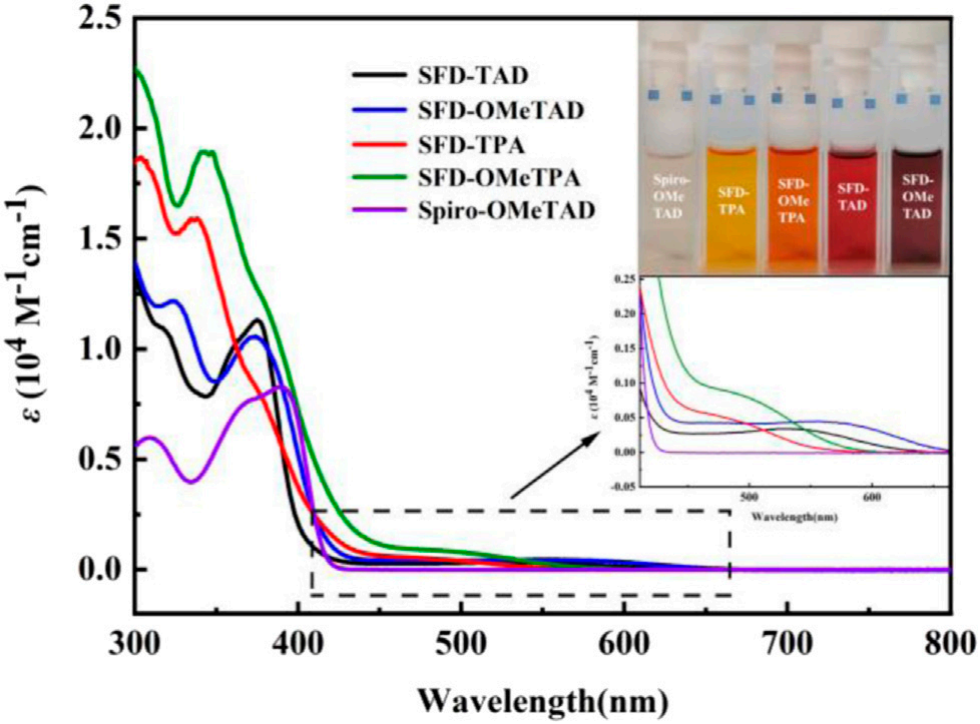
UV−vis absorption spectra and pictures of SFD-TPA, SFD-OMeTPA, SFD-TAD, and SFD-OMeTAD in CH_2_Cl_2_.

Optical properties. The spiro-OMeTAD dichloromethane solution is light yellow under sunlight, while the color of indenone spirofluorene cored products is deeper. UV-Vis absorption spectroscopy is used to study their optical properties in detail. As shown in Figure 2, these compounds display two bands in the range of 300–450 nm, which are assigned to n-π* transition and π−π* transition. Moreover, the bands in the range of 450–650 nm are observed obviously. This band is ascribed to the intramolecular charge transfer since there is no light absorption for spiro-OMeTAD and 2O-spiro (Xia et al., 2015). Furthermore, the UV-Vis absorption onset of diphenylamine moieties substituted indenone spirofluorene (SFD-TAD and SFD-OMeTAD) are in the longer wavelengths in comparison with triphenylamine substituted ones, indicating the direct linkage between 2O-spiro and N atoms favoring for effective charge transfer. The optical gaps of SFD-TPA, SFD-OMeTPA, SFD-TAD, and SFD-OMeTAD were calculated to be 2.22, 2.16, 1.97, and 1.87 eV, respectively, according to the formula, *E*_g_ = 1240/λ_onset_.

**FIGURE 3:**
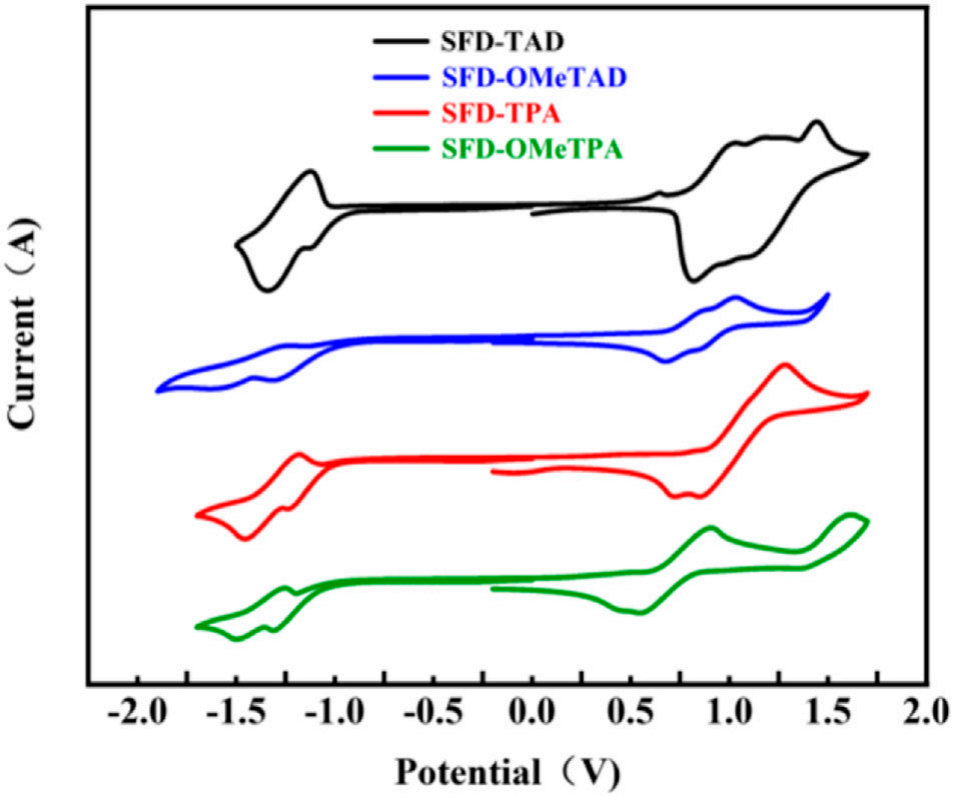
CV of SFD-TPA, SFD-OMeTPA, SFD-TAD, and SFD-OMeTAD.

Electrochemical properties. The electrochemical properties of SFD-TPA, SFD-OMeTPA, SFD-TAD, and SFD-OMeTAD were investigated by cyclic voltammetry (CV) in CH_2_Cl_2_ at a scan rate of 100 mV s^−1^. As shown in Figure 3, the CV of these four compounds displays reversible oxidation and reduction waves. Their reduction potentials are similar to that of the core 2O-spiro (Xia et al., 2015), and thus their lowest unoccupied molecular orbital (LUMO) energy levels are in the range of −3.24 to −3.35 eV. In comparison with the highest occupied molecular orbital (HOMO) energy level of spiro-OMeTPA, HOMO energy levels of SFD-TPA, SFD-OMeTPA, SFD-TAD, and SFD-OMeTAD decrease obviously, which is arising from the electron-withdrawing capability of carbonyl group from 2O-spiro. Moreover, with the onset voltage of the first oxidation potentials, the HOMO levels of SFD-TPA, SFD-OMeTPA, SFD-TAD, and SFD-OMeTAD were calculated to be −5.22, −4.96, −5.12, and −5.01 eV, respectively, according to the formula, *E*_*HOMO*_ = -[*E* ^*Ox*^+4.80-*E*_*(Fc/Fc+)*_], all data are exhibited in Table 1. On the basis of these results, it is predictable that methoxy groups favor the HOMO level enhancement. Consequently, the HOMO values for SFD-TPA, SFD-OMeTPA, and spiro-OMeTAD properly match with the valence band edge of the perovskite, leading to an effective hole extraction from the HTM, but also an efficient electron-blocking due to the high LUMO level. To further obtain a greater understanding of the geometric structure, electron distribution and frontier orbital energy levels of SFD-TPA, SFD-OMeTPA, SFD-TAD, and SFD-OMeTAD, density functional theory (DFT) calculations are carried out at the B3LYP/6-31G level. As shown in Supporting Information (Supplementary Figure S1), the electron density of LUMO distribution is mainly on the central core, while HOMO energy levels are almost delocalized across the whole molecule skeleton, which is similar to spiro-OMeTAD. The DSC curves of four target compounds are provided in Supplementary Figure S24.

**TABLE 1:** The optical and parameters of the target products.

	λ_max_ (nm)	λ_onset_ (nm)	*E*_LUMO_ ^a^ (eV)	*E*_HOMO_ ^a^ (eV)	*E*_g_ ^a^ (eV)	Gap^b^ (eV)
SFD-TAD	529.3	627.8	−3.35	−5.12	1.77	1.97
SFD-OMeTAD	561.6	660.6	−3.27	−5.01	1.74	1.87
SFD-TPA	457.7	557.4	−3.29	−5.22	1.93	2.22
SFD-OMeTPA	488.0	573.8	−3.24	−4.96	1.72	2.16
Spiro-OMeTAD	390.1	418.2	--	−4.72	--	2.96

a Obtained by CV curves.

b Obtained by UV-Vis absorption spectra.

**FIGURE 4:**
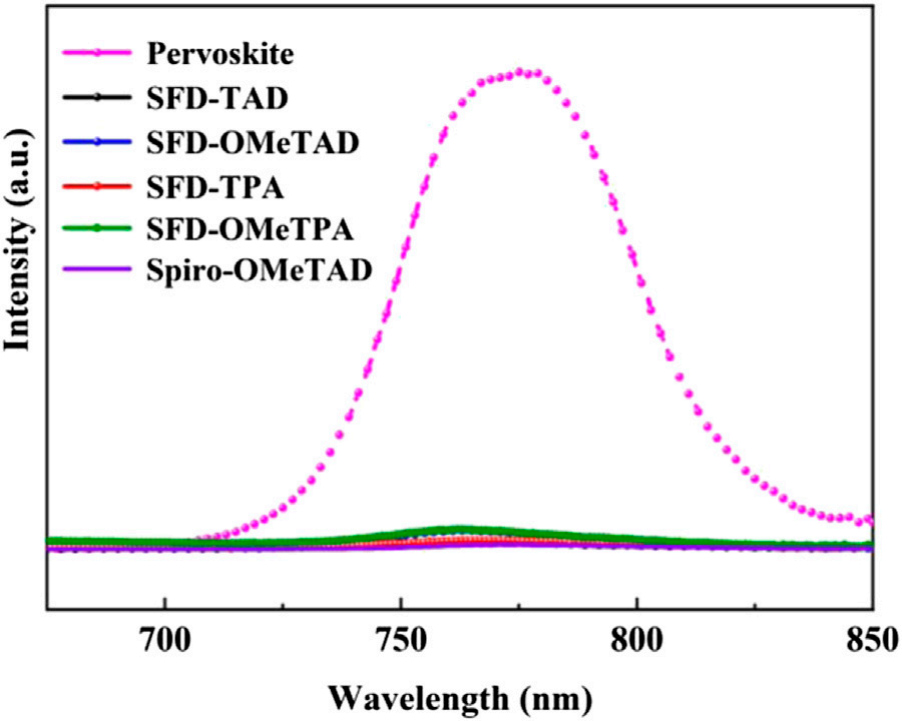
Steady-state photo luminance spectra of perovskite film and perovskite/HTM films.

To investigate the photoinduced charge transfer and charge separation between novel HTMs and perovskite, the photoluminescence (PL) quenching experiments were carried out (Figure 4). Compared with the bare perovskite film, when HTMs were introduced, the PL response of the pristine perovskite film was significantly quenched. This result indicates effective hole extraction and transport from perovskite to HTMs, and the quenching extent for four novel HTMs is at the same level of spiro-OMeTPA. Therefore, indenone spirofluorene cored molecules are proved to be promising novel D-A HTMs for PSCs. The novel D-A type hole-transporting materials with low cost for PSCs commercialization will be designed and synthesized in our laboratory.

## Conclusion

In summary, we have successfully constructed four novel D-A type indenone spirofluorene cored HTMs. The appropriate introduction of carbonyl groups into spiro-OMeTAD can not only lead intramolecular charge transfer effect but also modulate the frontier orbital energy levels. SFD-OMeTAD presents the optical gap as low as 1.87 eV, which significantly decreases in comparison with that (2.96 eV) of the reported spiro-OMeTAD. For the first time, we achieved the modification of classical spiro-OMeTAD into D-A type HTMs for perovskite solar cells.

## Data Availability

The datasets presented in this study can be found in online repositories. The names of the repository/repositories and accession number(s) can be found in the article/Supplementary Material.
