# Supplementary material for: The Evolution of Classical Spiro-OMeTAD: Synthesis of Arylamine Endcapped Indenone Spirofluorene
Source: Front Chem. 2022 May 31;10:898320. doi: 10.3389/fchem.2022.898320 (PMC9193283; doi:10.3389/fchem.2022.898320)
Supplement: Supplementary file 1 [file DataSheet1.docx]

**The Evolution of Classical Spiro-OMeTAD: Synthesis of Arylamine Endcapped Indenone Spirofluorene**

Shihui Liu, Xiaoqing Yi, Hao Wang, Tao Ye, Kui Wang, Wei Cao, Jing Guan*, Ruiqing Fan, Yulin Yang, Sue Hao*, Debin Xia*

[guanjing@ems.hrbmu.edu.cn](mailto:guanjing@ems.hrbmu.edu.cn); [haosue@hit.edu.cn](mailto:haosue@hit.edu.cn); [xia@hit.edu.cn](mailto:xia@hit.edu.cn)

Table of Contents

[1. General Information 2](#_Toc93945670)

[2. DFTsimilation 3](#_Toc93945671)

[3. Experimental Section 4](#_Toc93945672)

[4. NMR spectra 9](#_Toc93945673)

[5. Maldi-TOF mass Spectra 16](#_Toc93945674)

[6. IR spectra 20](#_Toc93945675)

# 1. General Information

Materials were all available commercially and used without further purification if not mentioned specially. Air sensitive reactions were carried out under argon or nitrogen atmosphere. Column chromatography was performed using silica gel (200-300 mesh). Analytical thin-layer chromatography was performed using Huanghai HSGF 254 plates. NMR spectra were recorded on a Bruker AVANCEIIIHD500MHz spectrometer at 298 K using partially deuterated solvents as internal standards. The UV-vis absorption spectra of the HTMs in dichloromethane (DCM) solutions (1×10^-5^ mol L^-1^) were obtained from Agilent Cary 60 using a 1 cm cuvette and recorded in the 200-800 nm wavelength range at room temperature. Cyclic voltammetry (CV) measurements were conducted using the Gamry interface 1000E electrochemical workstation with glassy carbon as the working electrode, platinum wire as the counter electrode, and Ag/AgCl electrode as the reference electrode in tetrabutylammonium hexafluorophosphate (n-Bu_4_NPF_6_, 0.1M) as supporting electrolyte with a scanning rate of 100mV/s at room temperature. The ferrocene/ferrocenium redox couple was applied as an external standard.

# 2. DFTsimilation


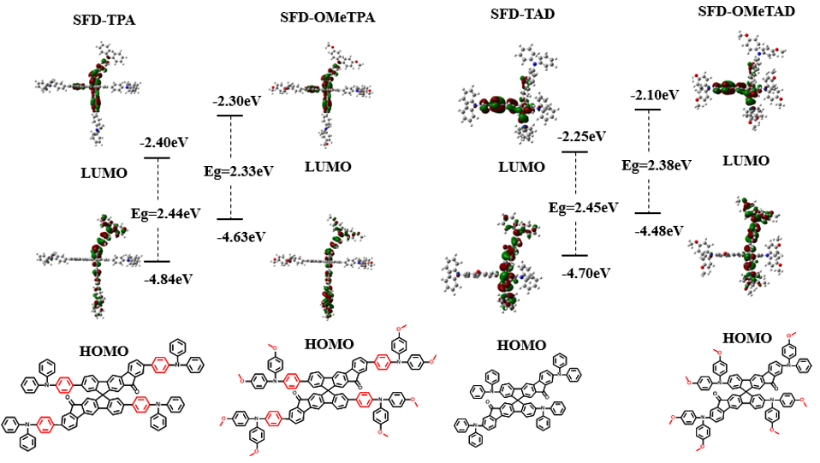


Figure S1. Energy diagram of frontier molecular orbitals computed at the B3LYP/6-31G level

# 3. Experimental Section

Scheme S1. Synthetic route to **SFD-TPA, SFD-OMeTPA, SFD-TAD** and **SFD-OMeTAD**.

***12H,12'H-10,10'-spirobi[indeno[2,1-b]fluorene]* (1) 2O-spiro** (1 g, 1.92 mmol) of dichloromethane solution (150 mL) was slowly added in 30 min to a stirred mixture of LiAlH_4_ (0.24 g, 6.15 mmol) and AlCl_3_ (1.62 g, 12.3 mmol) in 100 mL ether. The mixture was heated to 60°C under then reflux for 8 h. After the mixture cool to 0 ^o^C, water was added slowly to quench this reaction. The mixture was extracted with ethyl acetate, and the organic layer was washed with brine, and dried over anhydrous Na_2_SO_4_. The solvent was removed by rotary evaporation, and crude product was purified by column chromatography to afford a white solid in 72% yield. ^1^H NMR (500 MHz, CDCl_3_) δ 8.18 (s, 1H), 7.89 (d, J = 7.6 Hz, 1H), 7.85 (d, J = 7.6 Hz, 1H), 7.39 (d, J = 7.4 Hz, 1H), 7.34 (td, J = 7.5, 4.4 Hz, 2H), 7.21 (t, J = 7.4 Hz, 1H), 7.05 (t, J = 7.9 Hz, 1H), 6.85 (s, 1H), 6.72 (d, J = 7.5 Hz, 1H), 3.64 (s, 2H). ^13^C NMR (126 MHz, CDCl_3_) δ 149.31, 148.30, 143.65, 143.60, 141.90, 141.74, 141.50, 140.79, 127.77, 127.65, 126.77, 126.68, 125.02, 124.14, 120.83, 119.87, 119.79, 111.16, 65.59, 36.75. MALDI-TOF MS: m/z calcd for C_39_H_24_: 492.19; found: 492.2.

***2,2',8,8'-tetrabromo-12H,12'H-10,10'-spirobi[indeno[2,1-b]fluorene]* (2)** A three-neck round bottom flask (150 mL) equipped with a condenser and stirring bar, 120 mL of 1,1,2,2-tetrachloroethane (TCE) was added to dissolve 12H,12'H-10,10'-spirobi[indeno[2,1-b]fluorene](3) (1.0 g, 2.03 mmol). Br_2_ (1.6 g, 10.16 mmol) was diluted with TCE and add dropwise. The reaction mixture was stirred at room temperature for 12 h. Then, water was added. The mixture was extracted with ethyl acetate, and the organic layer was washed with Na_2_S_2_O_3_ solution, dried with anhydrous Na_2_SO_4_. The solvent was removed by rotary evaporation, and crude product was purified by column chromatography with eluent (petroleum ether: dichloromethane =1:15) to afford a white solid in 15% yield (0.24 g). ^1^H NMR (400 MHz, CDCl_3_) δ 8.91 (d, J = 8.0 Hz, 1H), 8.74 (d, J = 8.5 Hz, 1H), 7.62-7.55 (m, 2H), 7.47 (t, J = 7.7 Hz, 1H), 7.20 (t, J = 7.8 Hz, 1H), 6.83 (s, 1H), 6.78 (d, J = 7.6 Hz, 1H), 3.72 (s, 2H). MALDI-TOF Mass: m/z calcd for C_39_H_20_Br_4_: 807.83; found: 807.8238.

***2,2',8,8'-tetrabromo-12H,12'H-10,10'-spirobi[indeno[2,1-b]fluorene]-12,12'-dione* (3)** At room temperature, 100 mL two-necked flask equipped with an electromagnetic stirrer and an air condenser were charged compound **2** (0.5 g, 0.62 mmol), KOH (0.07 g, 1.24 mmol) and 10 mL THF. After 8 h stirring, mixture was filtered to remove KOH and the filtrate was concentrated to obtain the crude product, which was purified by column chromatography with the eluent (petroleum ether: dichloromethan1:7) to afford a white solid in 47% yield (0.25 g). ^1^H NMR (400 MHz, CDCl_3_) δ 8.91 (d, *J* = 8.1 Hz, 1H), 8.50 (d, *J* = 8.2 Hz, 1H), 7.78-7.65 (m, 2H), 7.52 (t, *J* = 8.1 Hz, 1H), 7.30 (d, *J* = 7.4 Hz, 1H), 6.96 (s, 1H), 6.82(d, *J* = 7.8 Hz, 1H). MALDI-TOF MS: m/z calcd for C_39_H_16_Br_4_O_2_: 835.78; found: 835.7900.

***2,2',8,8'-tetrakis(4-(diphenylamino)phenyl)-12H,12'H-10,10'-spirobi[indeno[2,1-b]fluorene]-12,12'-dione* (SFD-TPA)** In a schlenk tube, a mixture of **3** (200 mg, 0.239 mmol), N,N-diphenyl-4-(4,4,5,5-tetramethyl-1,3,2-dioxaborolan-2-yl)aniline (532.5 mg, 1.434 mmol), aqueous Na_2_CO_3_ 2M (2.4 mL) and Pd(PPh_3_)_4_ (27.7 mg, 0.024 mmol) in THF (8 mL) was refluxed overnight under inert atmosphere. After cooling to room temperature, the organic solvent was removed under vacuum and the solid dissolved in CH_2_Cl_2_. The aqueous phase was extracted with CH_2_Cl_2_ (2×10 mL) and the collected organic phases were washed with water (1×30 mL), and dried over Na_2_SO_4_. After removing the solvent under vacuum, the product was purified by column chromatography over silica gel using the eluent (petroleum ether:CH_2_Cl_2_ = 1:1) to give the product as red solid (142.7 mg, 40%).^1^H NMR (400 MHz, CDCl_3_) δ 7.83 (d, *J* = 1.8 Hz, 1H), 7.59 (dd, *J* = 8.4, 2.0 Hz, 1H), 7.49 (dp, *J* = 8.0, 3.3, 2.9 Hz, 5H), 7.41 (t, *J* = 7.9 Hz, 5H), 7.35 (s, 3H), 7.33 (s, 1H), 7.31 (s, 1H), 7.29 (s, 2H), 7.21-7.11 (m, 11H), 7.06 (t, *J* = 7.3 Hz, 3H), 6.92 (d, *J* = 7.7 Hz, 1H), 6.87 (d, *J* = 7.4 Hz, 1H), 6.65 (d, *J* = 8.0 Hz, 1H). ^13^C NMR (151 MHz, CD_2_Cl_2_) δ 192.12, 148.15, 146.81, 146.69, 140.09, 133.49, 132.32, 132.14, 131.22, 129.19, 129.00, 128.73, 128.53, 128.00, 127.13, 126.50, 124.08, 123.87, 123.39, 123.25, 123.15, 123.05, 122.80, 122.66, 122.52, 122.44, 120.79, 118.13, 28.92. MALDI-TOF MS: m/z calcd for C_111_H_72_N_4_O_2_: 1493.57; found: 1492.57.

***2,2',8,8'-tetrakis(4-(bis(4-methoxyphenyl)amino)phenyl)-12H,12'H-10,10'-spirobi[indeno[2,1-b]fluorene]-12,12'-dione* (SFD-OMeTPA)** In a schlenk tube, a mixture of **3** (200 mg, 0.239 mmol), 4-methoxy-N-(4-methoxyphenyl)-N-(4-(4,4,5,5-tetramethyl-1,3,2-dioxaborolan-2-yl)phenyl)aniline (619.5 mg, 1.434 mmol), aqueous Na_2_CO_3_ 2M (2.4 mL), Pd(PPh_3_)_4_ (27.7 mg, 0.024 mmol) in THF (8 mL) was refluxed overnight under inert atmosphere. After cooling to room temperature, the organic solvent was removed under vacuum and the solid dissolved in CH_2_Cl_2_. The aqueous phase was extracted with CH_2_Cl_2_ (2×10 mL) and the collected organic phases were washed with water (1×30 mL), and dried over Na_2_SO_4_. After removing the solvent under vacuum, the product was purified by column chromatography over silica using the eluent (petroleum ether:CH_2_Cl_2_ = 1:1) give the product as red solid (173.9 mg, 42%).^1^H NMR (500 MHz, CDCl_3_) δ 7.77 (s, 2H), 7.46 (t, J = 10.0 Hz, 4H), 7.41-7.35 (m, 6H), 7.27 (dd, J = 10.2, 3.7 Hz, 12H), 7.20-7.17 (m, 2H), 7.13 (d, J = 7.3 Hz, 2H), 7.08 (dd, J = 8.6, 3.2 Hz, 10H), 6.97-6.90 (m, 14H), 6.84 (dd, J = 8.7, 3.7 Hz, 10H), 6.61 (dd, J = 7.9, 3.7 Hz, 2H), 3.85 (d, J = 3.7 Hz, 12H), 3.80 (d, J = 3.9 Hz, 12H). ^13^C NMR (151 MHz, CD_2_Cl_2_) δ 193.63, 156.10, 156.00, 149.20, 148.95, 148.78, 146.86, 143.29, 142.47, 141.12, 140.98, 140.65, 135.85, 134.17, 132.96, 131.82, 129.72, 129.43, 128.91, 128.72, 127.90, 127.05, 126.89, 126.77, 124.09, 123.97, 123.53, 121.67, 120.94, 120.77, 120.27, 119.06, 114.88, 114.70, 55.52, 55.47, 26.04, 25.90. MALDI-TOF MS: m/z calcd for C_119_H_88_N_4_O_10_: 1732.65; found: 1732.64.

***2,2',8,8'-tetrakis(bis(4-methoxyphenyl)amino)-12H,12'H-10,10'-spirobi[indeno[2,1-b]fluorene]-12,12'-dione* (SFD-TAD)** In a schlenk tube, a mixture of **3** (100 mg, 0.12 mmol), diphenylamine (121 mg, 0.72 mmol), *t*-BuONa (92 mg, 0.96 mmol), P (*t*-Bu)_3_ (1.22 mg, 0.006 mmol) and Pd(OAc)_2_ (11 mg, 0.048 mmol) in dry toluene was refluxed overnight under inert atmosphere. After cooling to room temperature, the organic solvent was removed under vacuum. The product was purified by column chromatography over silica gel using the eluent (petroleum ether:CH_2_Cl_2_ = 1:1) to give the product as black solid (54.2 mg, 38%). ^1^H NMR (500 MHz, CDCl_3_) δ 7.94 (d, *J* = 7.8 Hz, 2H), 7.45-7.35 (m, 15H), 7.32-7.26 (m, 5H), 7.26-7.21 (m, 11H), 7.16 (t, *J* = 7.7 Hz, 3H), 7.09-7.02 (m, 17H), 7.01-6.93 (m, 4H), 6.88 (dd, *J* = 8.4, 2.3 Hz, 2H), 6.64 (d, *J* = 7.5 Hz, 2H). ^13^C NMR(126 MHz, CDCl_3_) δ 192.60, 149.79, 149.16, 148.98, 148.58, 146.74, 145.64, 144.03, 143.84, 138.94, 136.71, 136.27, 134.78, 133.67, 133.62, 129.65, 129.51, 129.40, 129.31, 129.28, 128.57, 127.00, 125.24, 124.82, 124.65, 123.92, 123.41, 121.97, 121.74, 119.42, 119.40, 118.81, 117.65, 115.28, 77.26, 77.01, 76.75, 65.92, 53.42. MALDI-TOF MS:: m/z calcd for C_87_H_56_N_4_O_2_: 1188.44; found: 1188.4.

***2,2',8,8'-tetrakis(diphenylamino)-12H,12'H-10,10'-spirobi[indeno[2,1-b]fluorene]-12,12'-dione* (SFD-OMeTAD)** In a schlenk tube, a mixture of **3** (100 mg, 0.12 mmol), 4,4’-dimethoxy-4-biphenylamin (165 mg, 0.72 mmol), *t*-BuONa (92 mg, 0.96 mmol), P (*t*-Bu)_3_ (1.22 mg, 0.006 mmol) and Pd(OAc)_2_ (11 mg, 0.048 mmol) in dry toluene was refluxed overnight under inert atmosphere. After cooling to room temperature, the organic solvent was removed under vacuum. The product was purified by column chromatography over silica gel using the eluent (petroleum ether:CH_2_Cl_2_ = 1:1) to give the product as black solid (71.9 mg, 42%).^1^H NMR (500 MHz, CDCl_3_) δ 7.93 (d, *J* = 7.8 Hz, 2H), 7.37-7.26 (m, 9H), 7.25 (s, 2H), 7.16 (t, *J* =7.6 Hz, 2H), 7.11-7.05 (m, 5H), 7.04-6.95 (m, 11H), 6.90 (d, *J* = 9.1 Hz, 5H), 6.86-6.76 (m, 14H), 6.72 (dd, *J* = 8.4, 2.3 Hz, 2H), 6.63 (d, *J* = 7.5 Hz, 2H). ^13^C NMR(126 MHz, CDCl_3_) δ 156.52, 154.23, 154.09, 149.94, 149.14, 148.75, 139.76, 139.15, 138.09, 137.88, 136.69, 133.12, 129.11, 128.46, 127.28, 124.84, 124.70, 123.80, 123.38, 120.17, 119.12, 114.93, 114.80, 114.68. MALDI-TOF MS: m/z calcd for C_95_H_72_N_4_O_2_: 1428.52; found: 1428.5.

# 4. NMR spectra


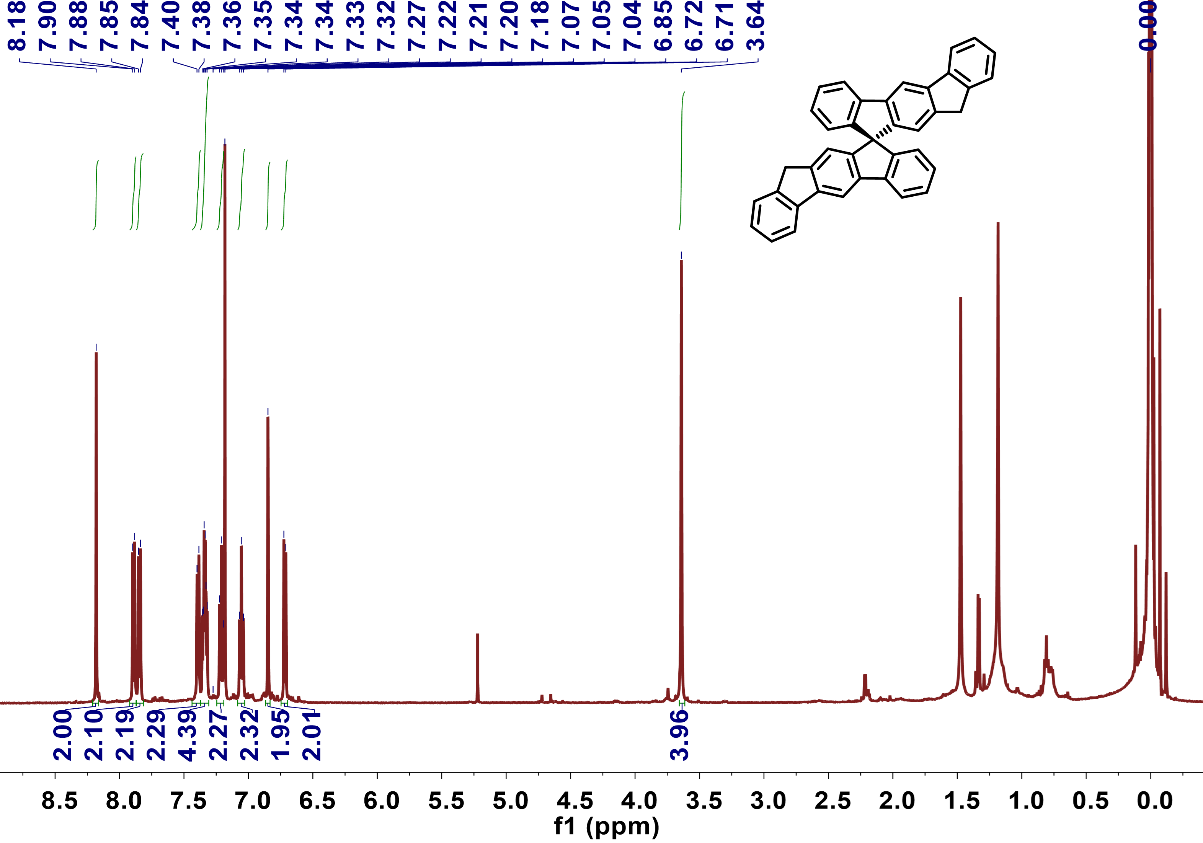


Figure S2. ^1^H NMR spectrum of compound **1** (500 MHz) in CDCl_3_, 298K


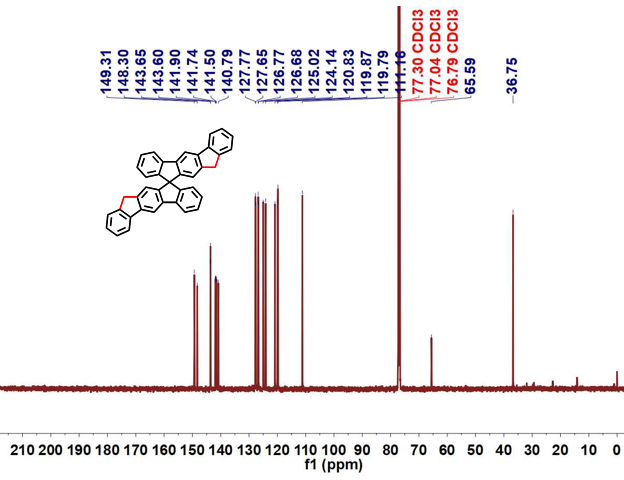


Figure S3. ^13^C NMR spectrum of compound **1** (126 MHz) in CDCl_3_, 298K


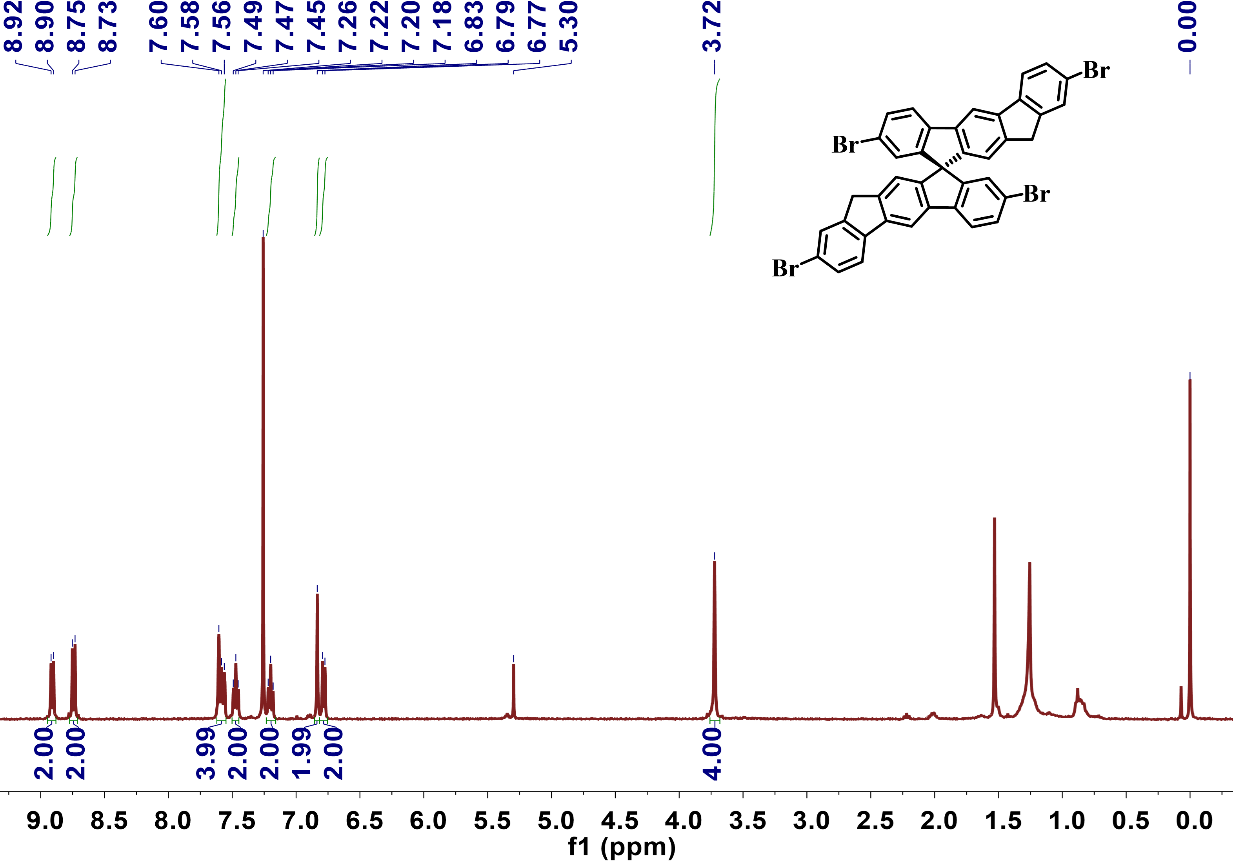


Figure S4. ^1^H NMR spectrum of compound **2** (400 MHz) in CDCl_3_, 298K


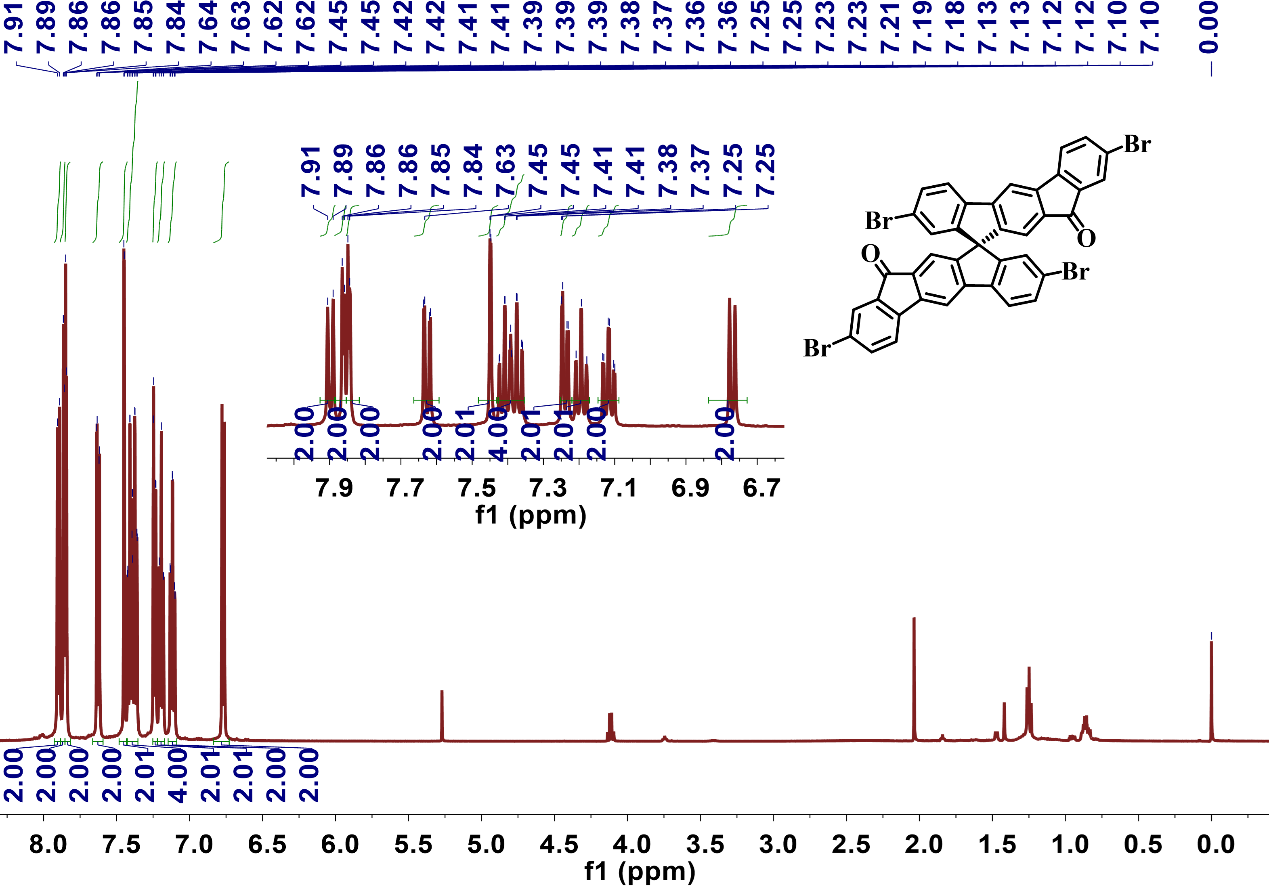


Figure S5. ^1^H NMR spectrum of compound **3** (400 MHz) in CDCl_3_, 298K


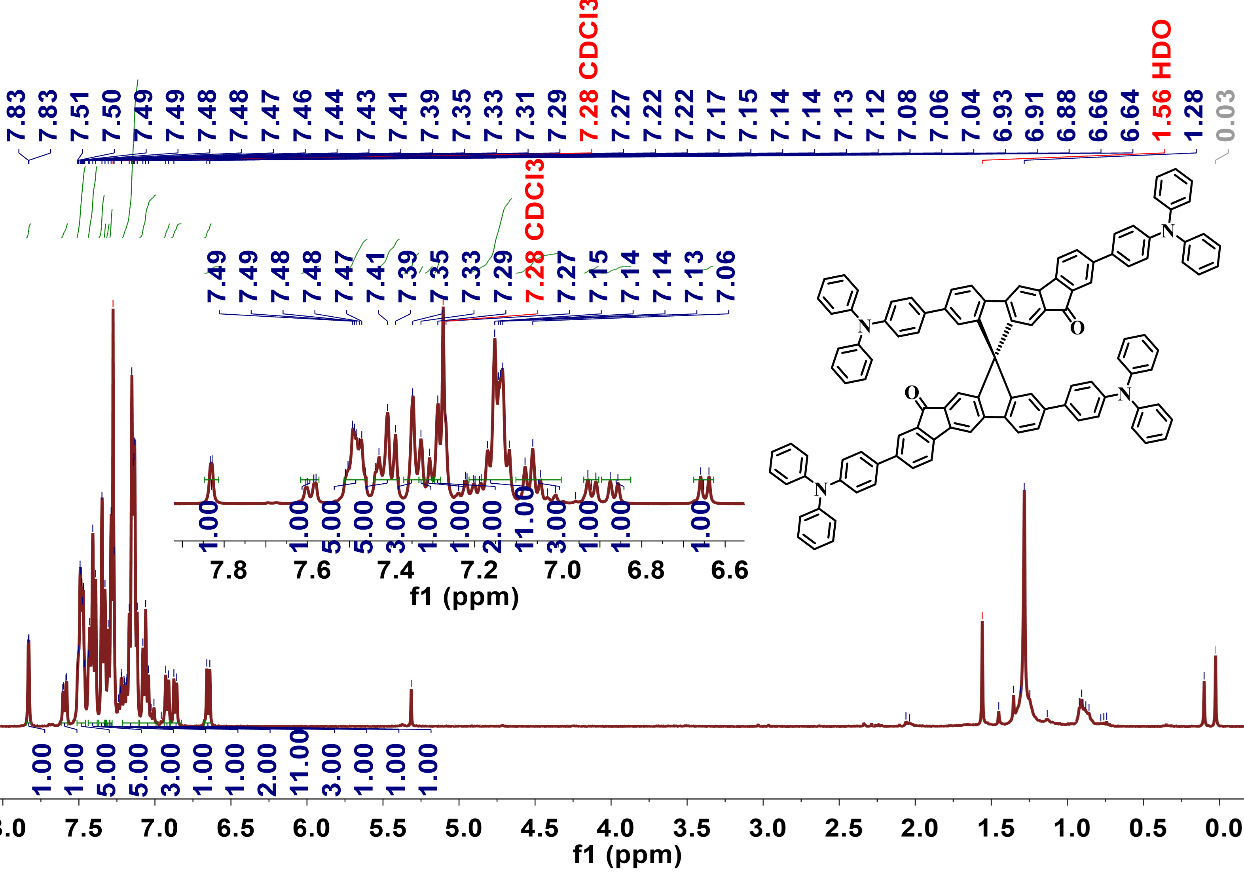


Figure S6. ^1^H NMR spectrum of **SFD-TPA** (400 MHz) in CDCl_3_, 298K


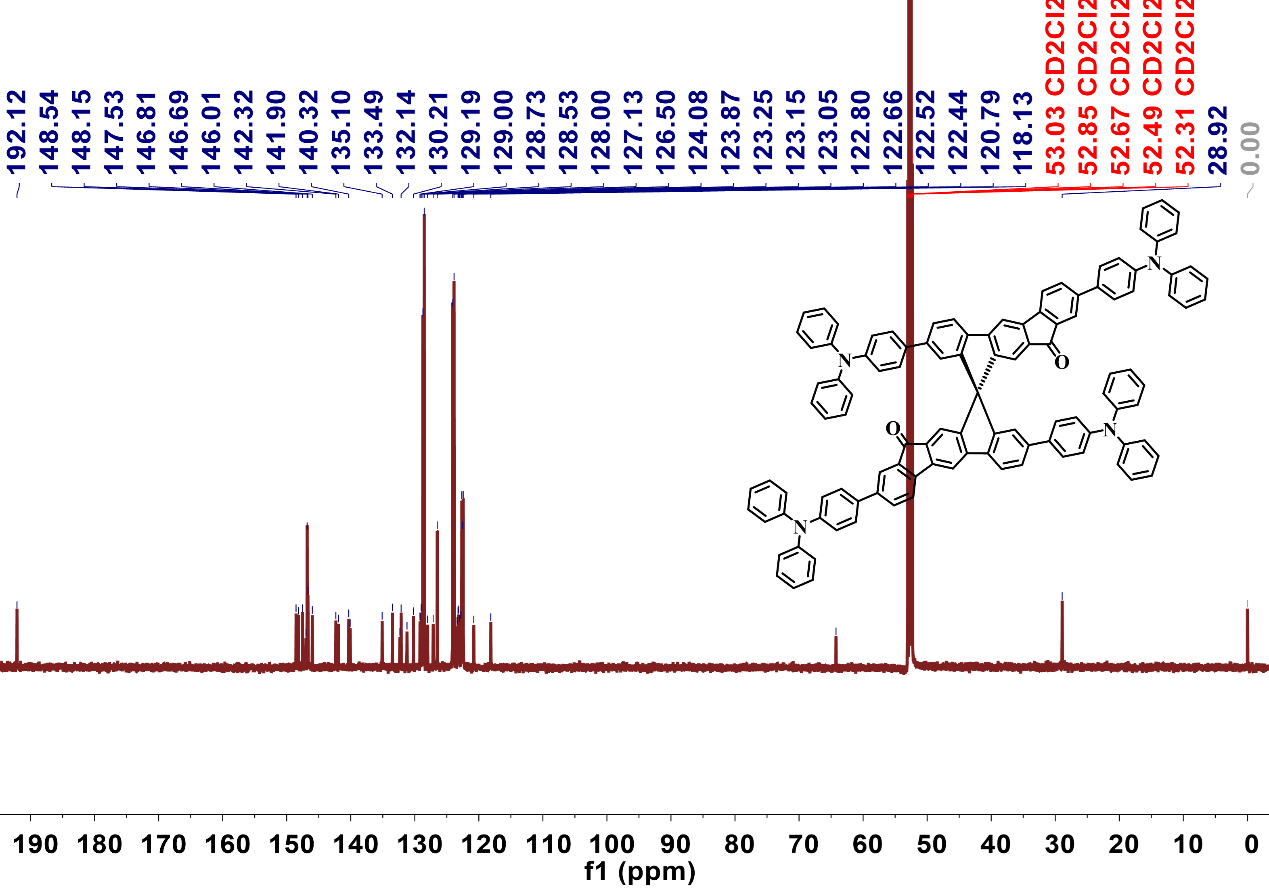


Figure S7. ^13^C NMR spectrum of **SFD-TPA** (125 MHz) in CDCl_3_, 298K


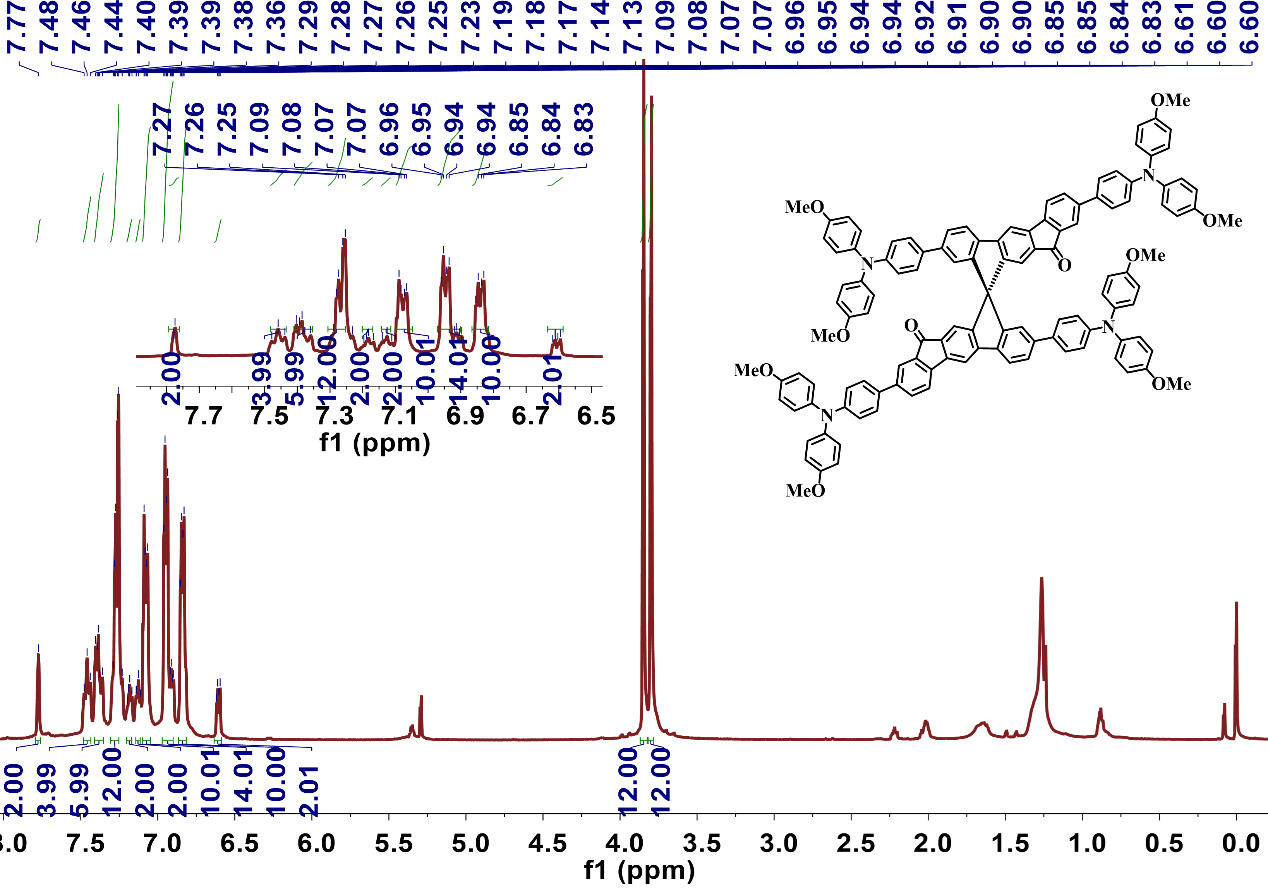


Figure S8. ^1^H NMR spectrum of **SFD-OMeTPA** (400 MHz) in CDCl_3_, 298K


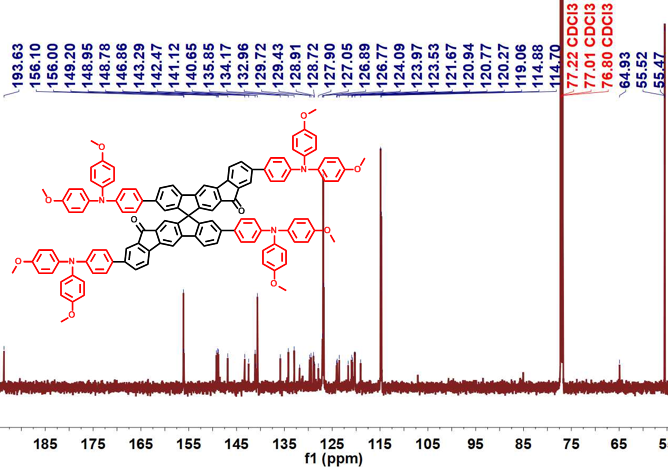

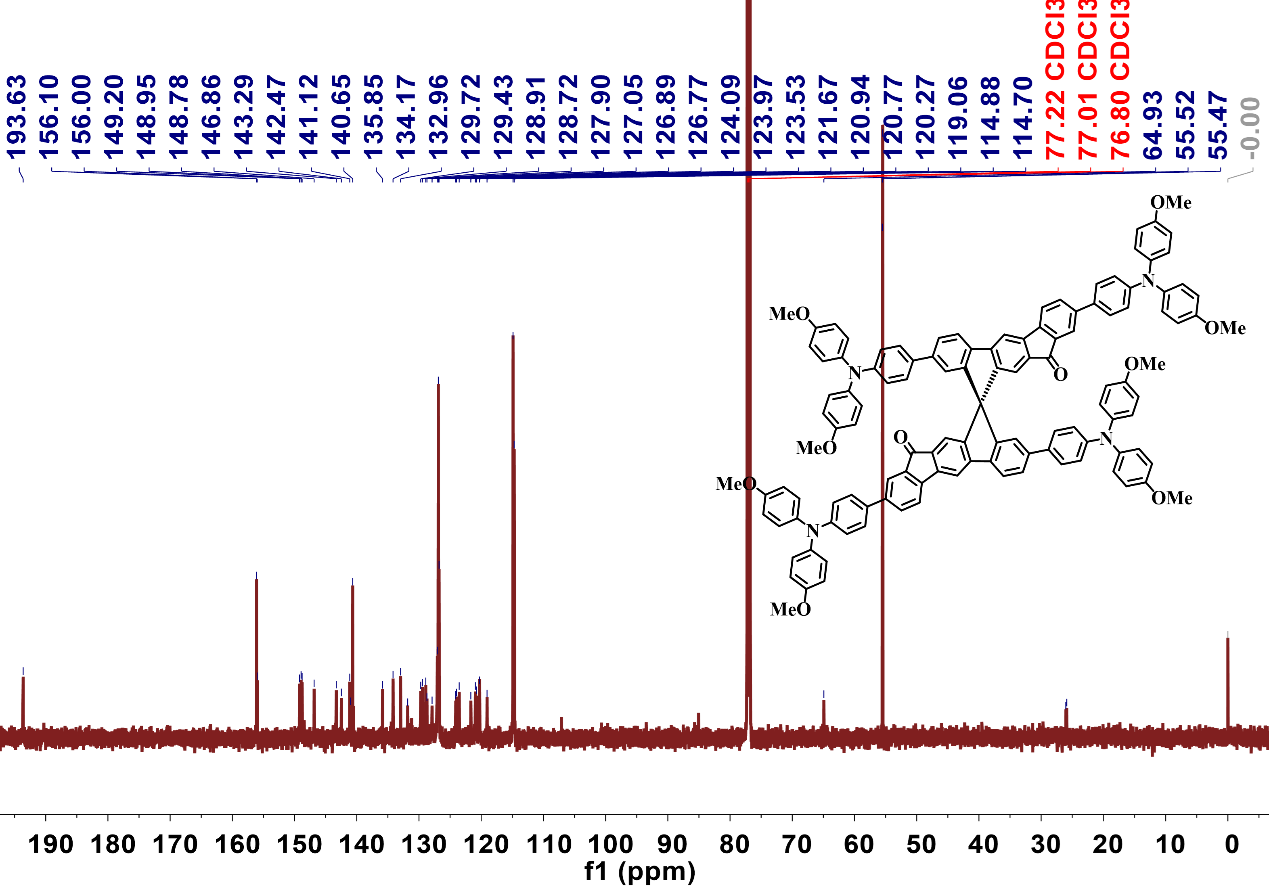


Figure S9. ^13^C NMR spectrum of **SFD-OMeTPA** (125 MHz) in CDCl_3_, 298K


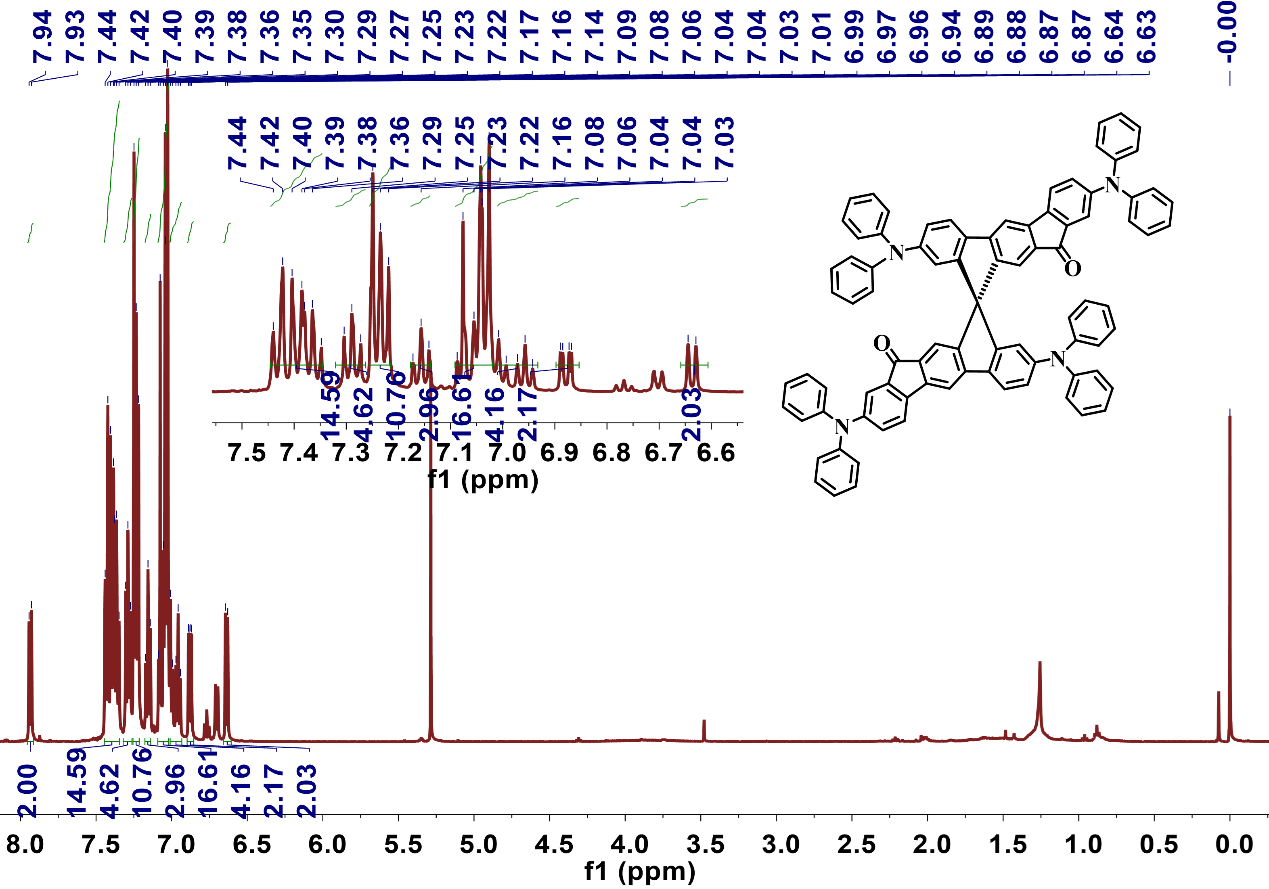


Figure S10. ^1^H NMR spectrum of **SFD-TAD** (500 MHz) in CDCl_3_, 298K


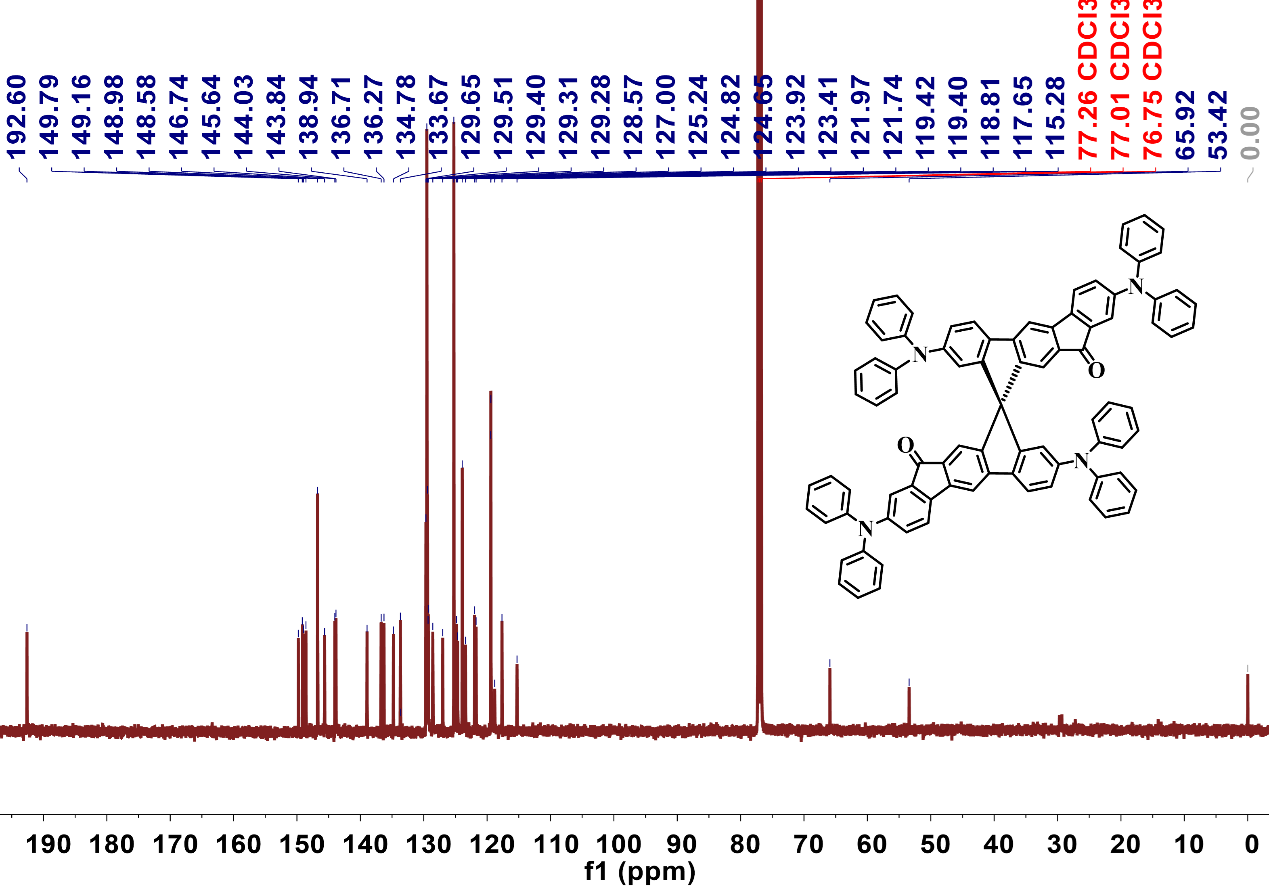


Figure S11. ^13^C NMR spectrum of **SFD-TAD** (125 MHz) in CDCl_3_, 298K


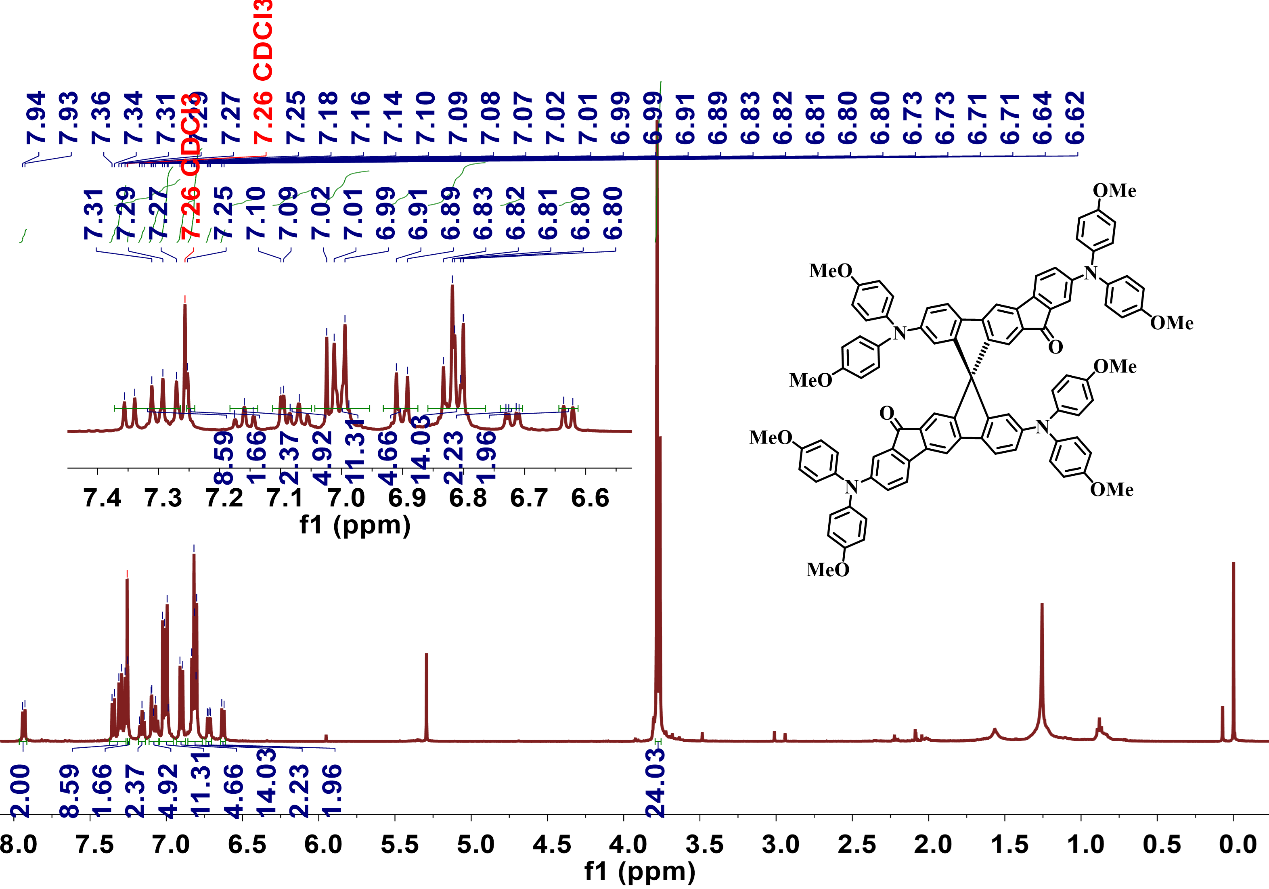


Figure S12. ^1^H NMR spectrum of **SFD-OMeTAD** (500 MHz) in CDCl_3_, 298K


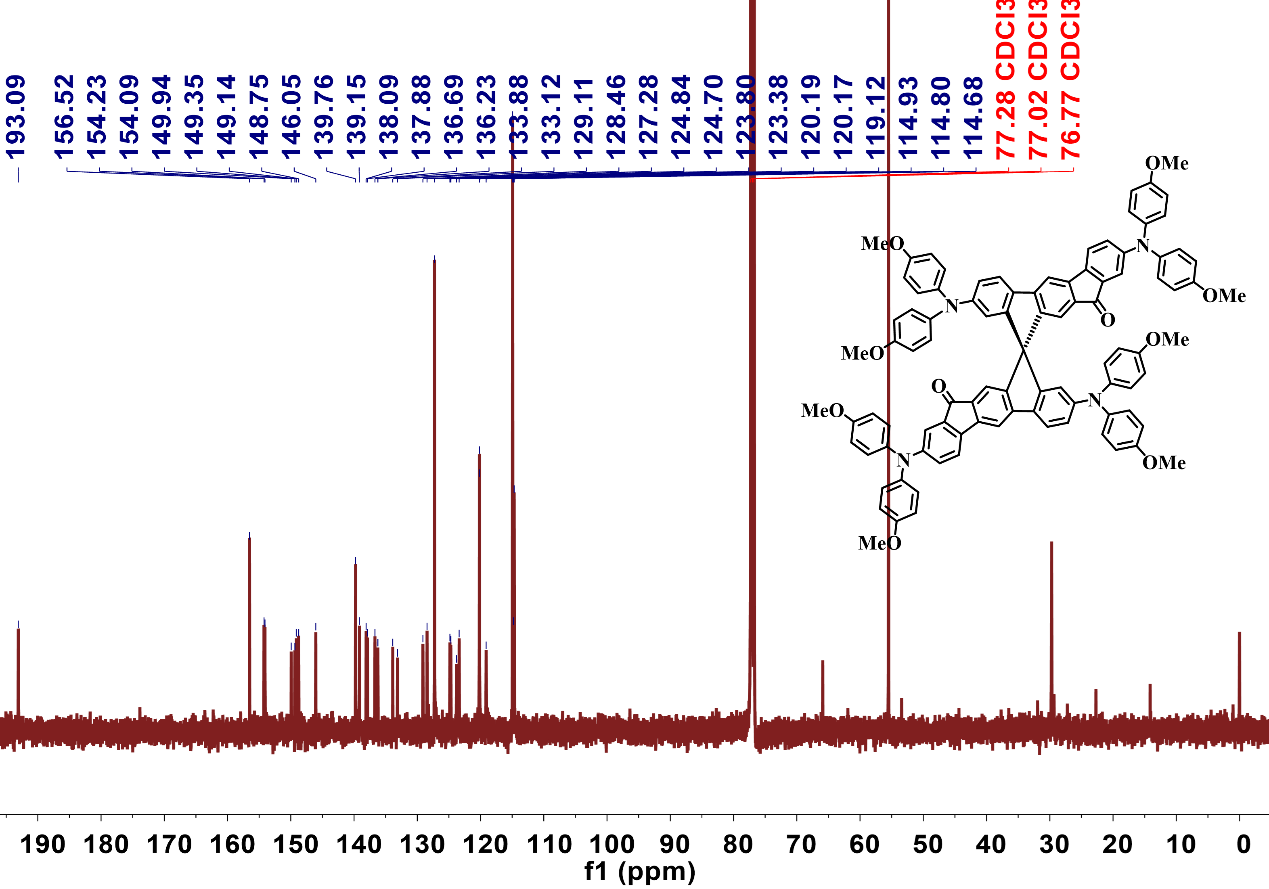


Figure S13. ^13^C NMR spectrum of **SFD-OMeTAD** (500 MHz) in CDCl_3_, 298K

# 5. Maldi-TOF mass Spectra


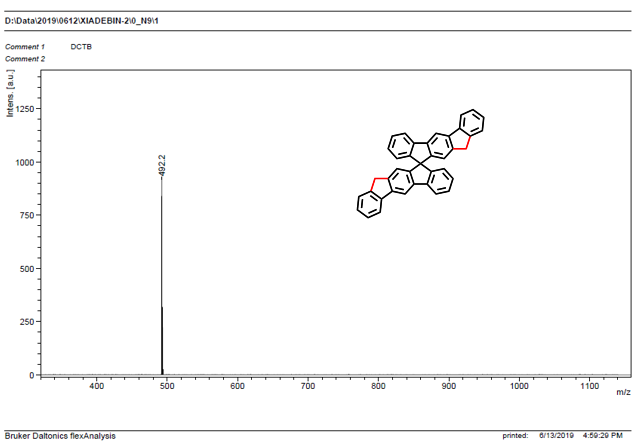


Figure S14. Maldi-TOF-Mass spectrum of compound **1**.


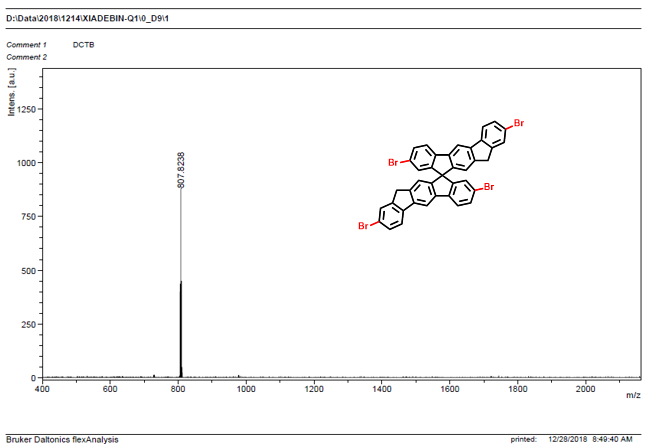


Figure S15. Maldi-TOF-Mass spectrum of compound **2**.


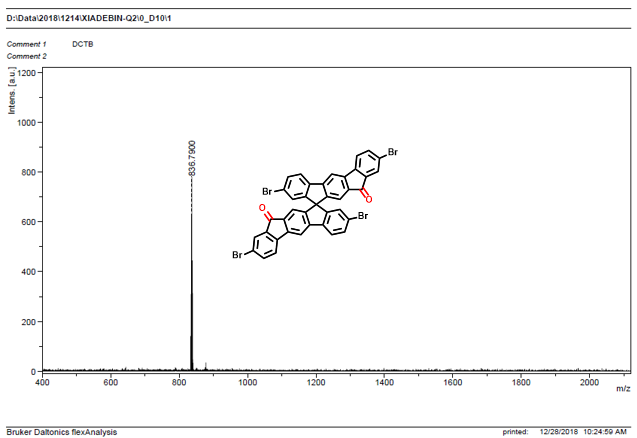


Figure S16. Maldi-TOF-Mass spectrum of compound **3**.


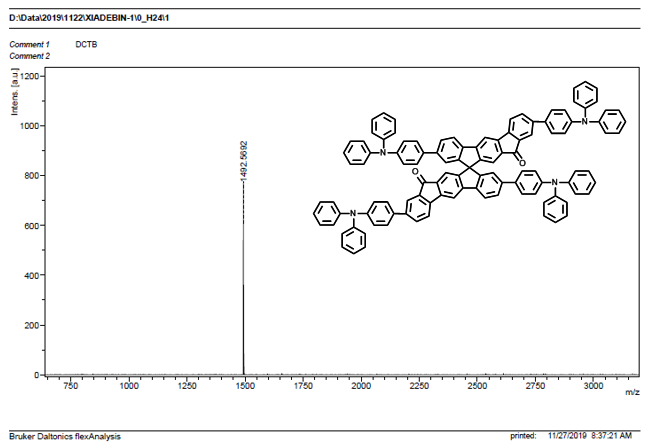


Figure S17. Maldi-TOF-Mass spectrum of **SFD-TPA**.


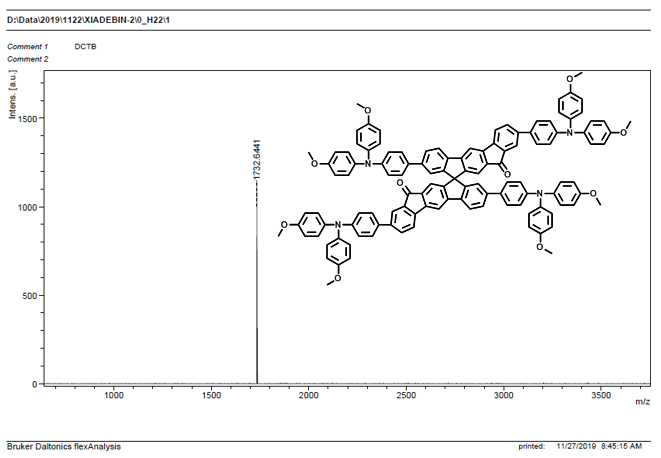


Figure S18. Maldi-TOF-Mass spectrum of **SFD-OMeTPA**.


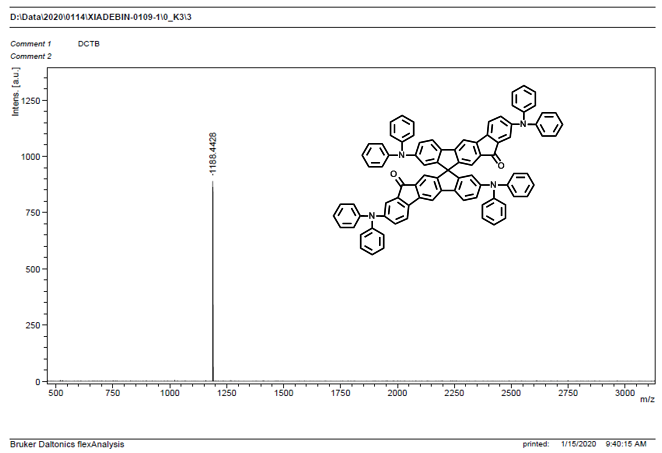


Figure S19. Maldi-TOF-Mass spectrum of **SFD-TAD**.


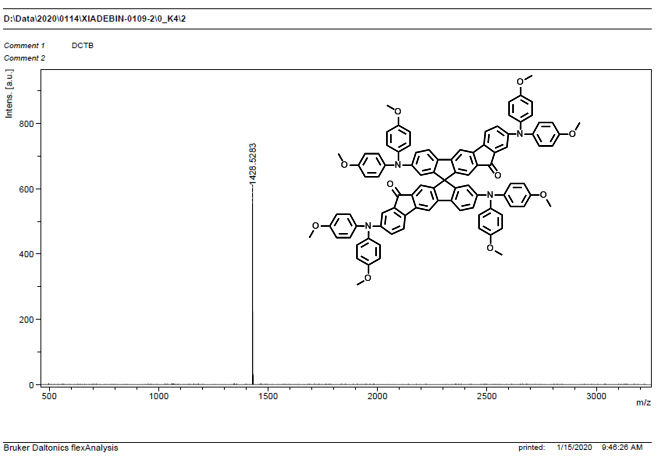


Figure S20. Maldi-TOF-Mass spectrum of **SFD-OMeTAD**.


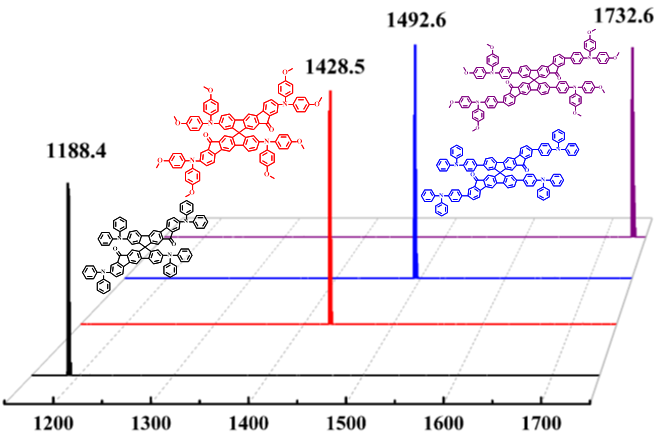


Figure S21. Maldi-TOF-Mass spectra of four target compounds.

# 6. IR spectra


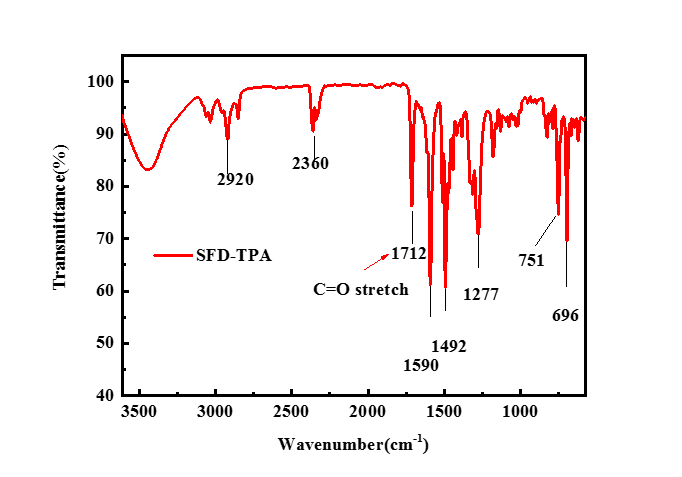


Figure S22. the IR spectra of **SFD-TPA**


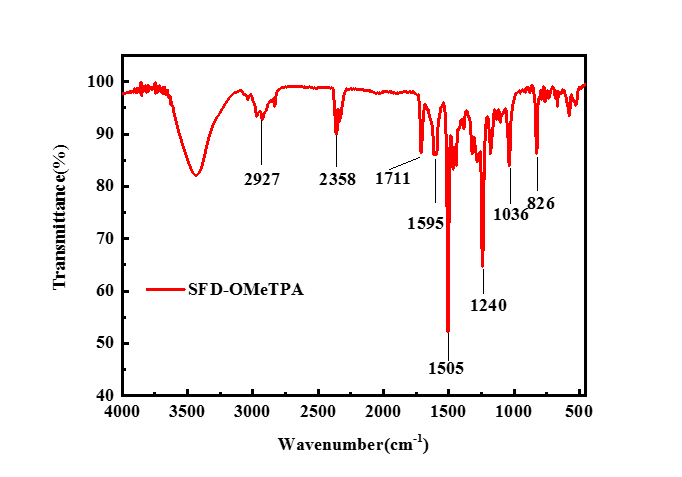


Figure S23. the IR spectra of **SFD-OMeTPA**

# 7. DSC spectra


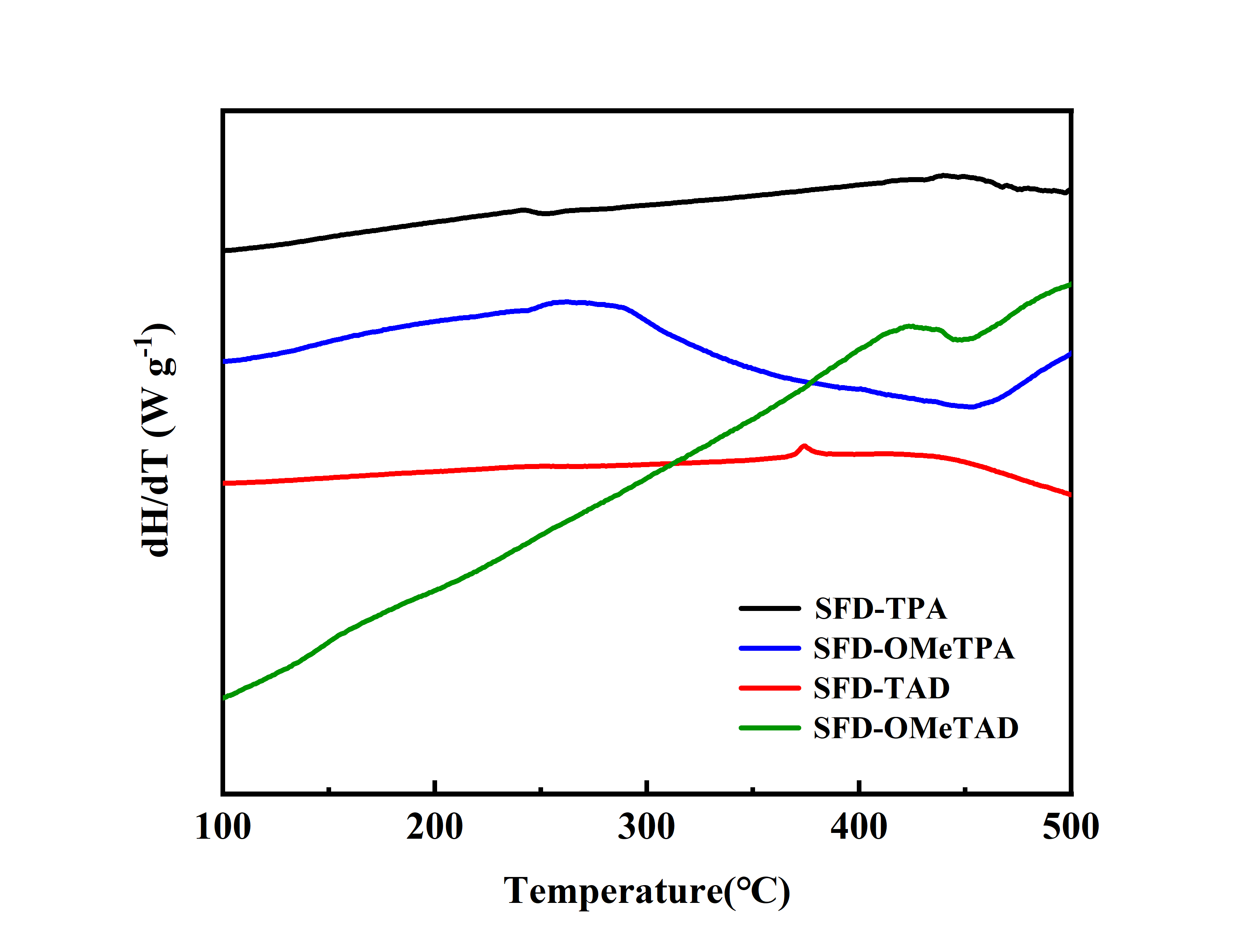


Figure S24. the DSC spectra of four target compounds

# 8. SCLC curve


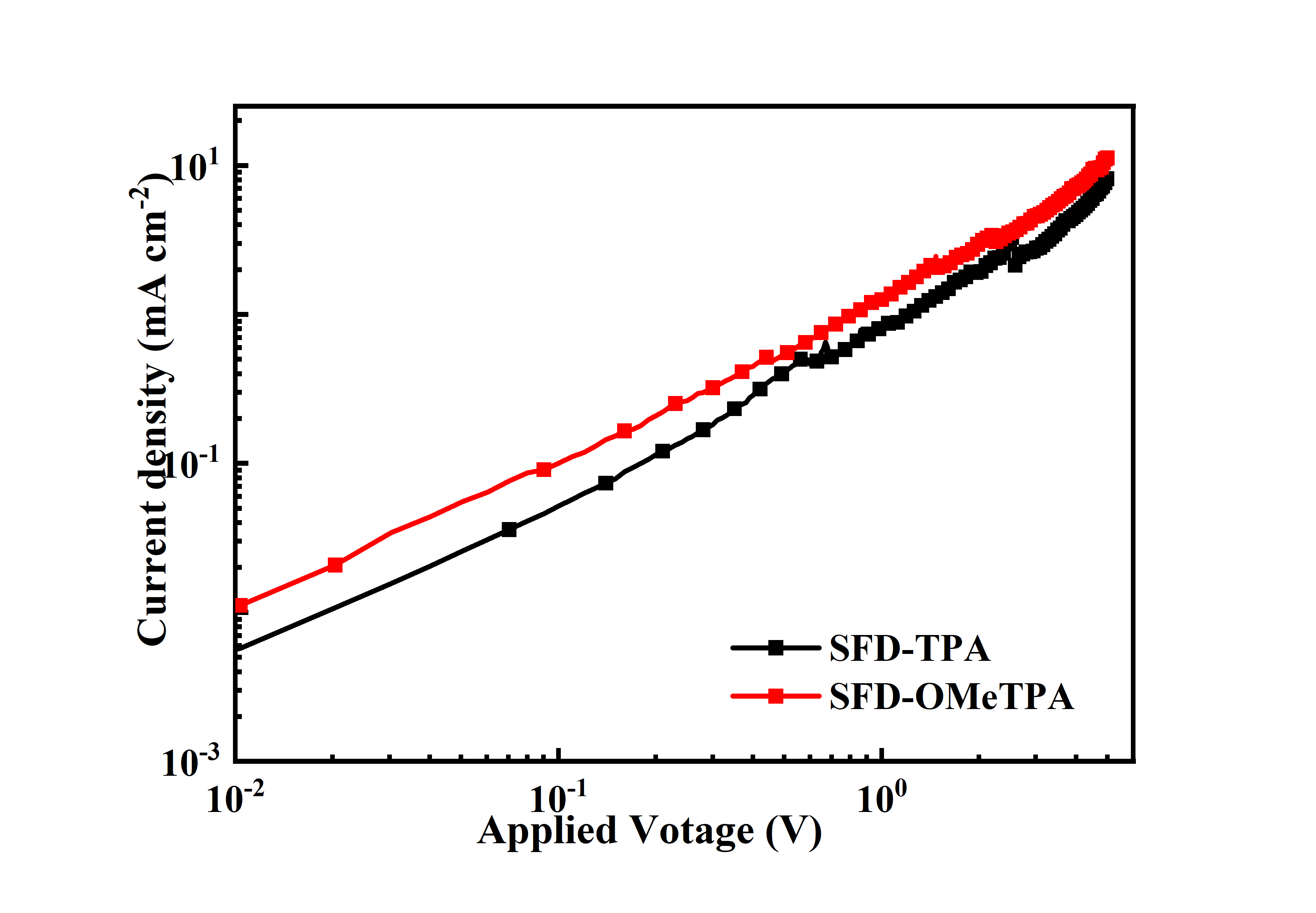


Figure S25. The dark J-V curves of SFD-TPA and SFD-OMeTPA based hole-only devices
